# Active and dormant microorganisms on glacier surfaces

**DOI:** 10.1111/gbi.12535

**Published:** 2022-11-30

**Authors:** James A. Bradley, Christopher B. Trivedi, Matthias Winkel, Rey Mourot, Stefanie Lutz, Catherine Larose, Christoph Keuschnig, Eva Doting, Laura Halbach, Athanasios Zervas, Alexandre M. Anesio, Liane G. Benning

**Affiliations:** ^1^ Queen Mary University of London London UK; ^2^ GFZ German Research Centre for Geosciences Berlin Germany; ^3^ Bundesanstalt für Risikobewertung (BfR) Berlin Germany; ^4^ Freie University Berlin Berlin Germany; ^5^ Environmental Microbial Genomics Université de Lyon Ecully Cedex France; ^6^ Environmental Science Aarhus University Roskilde Denmark

**Keywords:** activity, dormancy, extremophiles, glacier, ice, microorganisms, snow

## Abstract

Glacier and ice sheet surfaces host diverse communities of microorganisms whose activity (or inactivity) influences biogeochemical cycles and ice melting. Supraglacial microbes endure various environmental extremes including resource scarcity, frequent temperature fluctuations above and below the freezing point of water, and high UV irradiance during summer followed by months of total darkness during winter. One strategy that enables microbial life to persist through environmental extremes is dormancy, which despite being prevalent among microbial communities in natural settings, has not been directly measured and quantified in glacier surface ecosystems. Here, we use a combination of metabarcoding and metatranscriptomic analyses, as well as cell‐specific activity (BONCAT) incubations to assess the diversity and activity of microbial communities from glacial surfaces in Iceland and Greenland. We also present a new ecological model for glacier microorganisms and simulate physiological state‐changes in the glacial microbial community under idealized (i) freezing, (ii) thawing, and (iii) freeze–thaw conditions. We show that a high proportion (>50%) of bacterial cells are translationally active in‐situ on snow and ice surfaces, with Actinomycetota, Pseudomonadota, and Planctomycetota dominating the total and active community compositions, and that glacier microorganisms, even when frozen, could resume translational activity within 24 h after thawing. Our data suggest that glacial microorganisms respond rapidly to dynamic and changing conditions typical of their natural environment. We deduce that the biology and biogeochemistry of glacier surfaces are shaped by processes occurring over short (i.e., daily) timescales, and thus are susceptible to change following the expected alterations to the melt‐regime of glaciers driven by climate change. A better understanding of the activity of microorganisms on glacier surfaces is critical in addressing the growing concern of climate change in Polar regions, as well as for their use as analogues to life in potentially habitable icy worlds.

## 1 INTRODUCTION

Glaciers and ice sheets contain a large reservoir of microbial life (Anesio et al., 2017), whose activity plays a role in Earth's biogeochemical cycles (Wadham et al., 2019). Microorganisms on the surfaces of glaciers can also contribute to the darkening of the ice surface and enhance ice melt, consequently affecting sea level rise (Benning et al., 2014; Cook et al., 2020; Lutz et al., 2016; Williamson et al., 2020). High‐latitude regions are distinctly seasonal, and thus glacier surfaces are commonly characterized by continuous sunlight during summer, and months of complete darkness and freezing temperatures during winter. One strategy that enables microbial life to persist throughout extended periods of unfavourable conditions is dormancy – a reversible state of low metabolic activity. Dormancy is prevalent among microorganisms in natural settings (Lennon & Jones, 2011), with over half of all microorganisms in freshwater habitats and over two‐thirds of microorganisms in soils thought to be in a metabolically inactive state at any one time (Jones & Lennon, 2010). Repeated transitions into and out of dormancy have been shown to enable high levels of microbial biodiversity to be maintained over time (Jones & Lennon, 2010; Shoemaker & Lennon, 2017). Glacier surfaces are highly dynamic ecosystems, that pose various challenges to their inhabitants, including temperature fluctuations above and below the freezing point of water (across multiple timescales, i.e., hourly to seasonally), resource scarcity, and light (high UV irradiance during summer and total darkness during winter). Dormancy, therefore, may be especially critical to the survival of glacial microorganisms. However, the prevalence of dormancy among glacier surface‐dwelling microorganisms in high‐latitude glacial habitats, its relationship to environmental conditions, and its biogeochemical and evolutionary implications have not been extensively studied.

The Arctic is currently experiencing striking changes to its climate, including significant warming and expansion of glacier ablation zones (Noël et al., 2019). Climate change in the Arctic is intensified by the Polar amplification effect, and the Arctic is warming at three to four times the global average rate (AMAP, 2021; Rantanen et al., 2022). It is expected that future decades will be characterized by both a general increase in average air temperatures in the Arctic and an increased frequency and intensity of winter warming (Graham et al., 2017; Post et al., 2019). In addition, the springtime melting of snow and ice will occur earlier (Assmann et al., 2019), and phenological events (including the heightening of biological activity) will arrive earlier (Post et al., 2018). Put simply, the duration and extent of melt across the surface of Arctic glaciers and ice sheets, as well as the development of microbial blooms that further enhance this melt, are increasing as anthropogenic climate warming continues.

The lengthening of the melt‐season, as well as the increased frequency and intensity of warming and rain events, especially during winter (Doyle et al., 2015; Førland et al., 2020), have the potential to significantly alter the glacial microbiome. Microbial activity is critically dependent on the availability of liquid water (Price, 2007), and thus it follows that any changes to glacier surface melt could drastically affect the activity of surface‐ice dwelling microbial communities, potentially altering the functioning of glacial ecosystems and associated biogeochemical cycles. Nevertheless, the response time of microbial activity to the onset of melt on glacier surfaces is not well known. This is a critical parameter in determining how susceptible glacial microorganisms, their activity, and associated biogeochemical cycles might be to future climate change.

**FIGURE 1:**
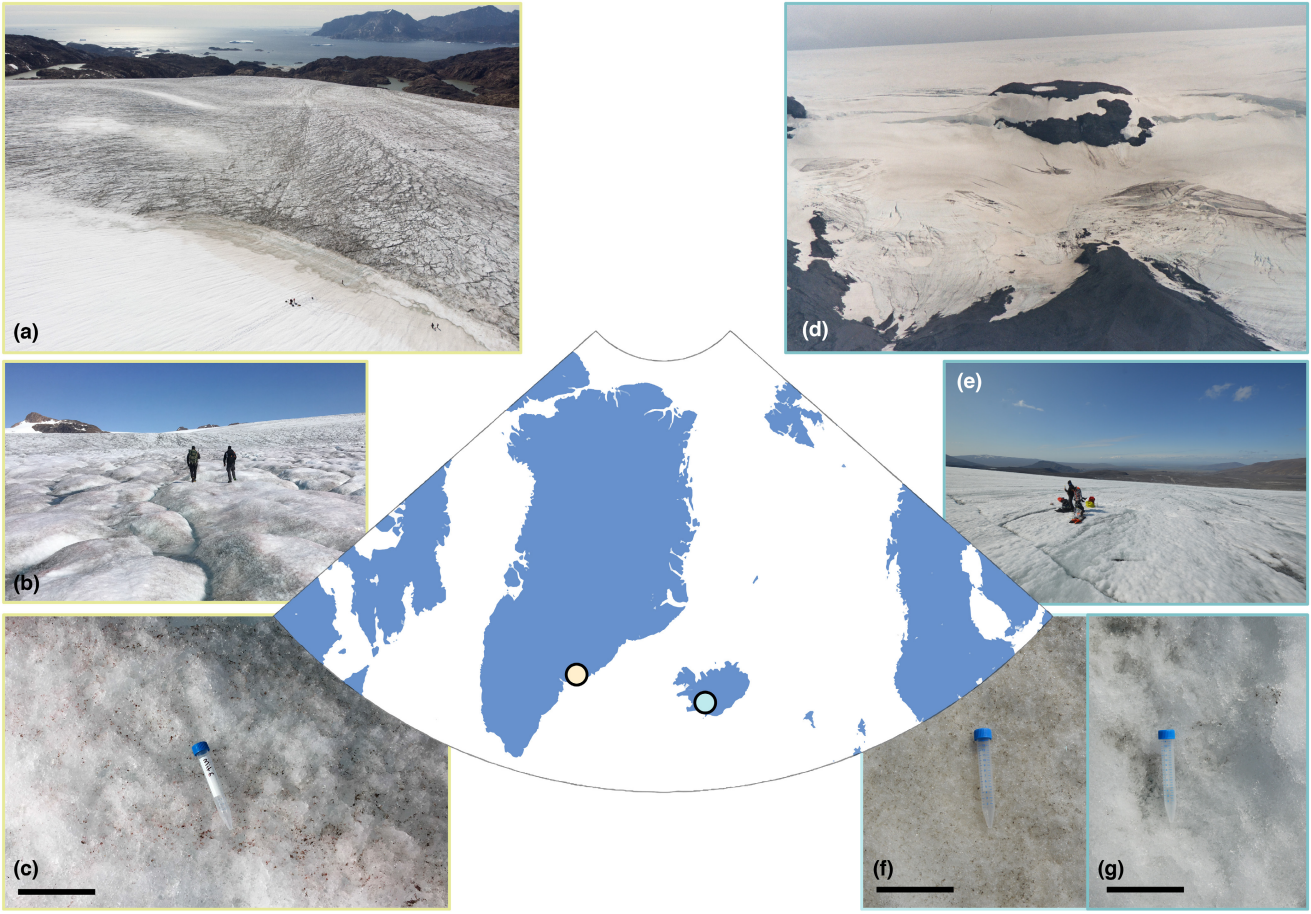
Field sites. (a–c) Mittivakkat glacier in SE‐Greenland; (a) the transition from the snow to the ice surface; (b) the glacier surface, and (c) a close‐up of the ice surface. (d–g) Langjökull, Iceland, (d) as seen from Kaldadalsvegur (credit: Johann Dréo, CC BY‐SA 3.0); (e) the Langjökull glacier surface; and close‐ups of the (f) snow and (g) ice surface. The scale bars in c, f, and g represent 10 cm.

Here, we present results from a study of microbial community dynamics on two glacier surfaces: Langjökull in Iceland and Mittivakkat glacier in SE‐Greenland (Figure 1). We used bioorthogonal non‐canonical amino acid tagging (BONCAT) to detect translationally active (i.e., protein synthesis‐active) cells (Hatzenpichler et al., 2014) and quantified the active and dormant fractions of the snow and ice‐dwelling communities in‐situ during the summer melt season. We also used BONCAT in ex‐situ incubations following the thawing of snow and ice samples that were preserved in a frozen state (−20°C) for 6 months (approximating winter conditions). Further, we analyzed the nutrient dynamics and characterized the diversity of microbial communities on the glacier snow and ice surfaces using 16S and 18S rRNA gene sequences and compared this to the diversity of the active consortia of bacteria and eukaryotes as determined by full‐length 16S and 18S rRNA gene sequences extracted from total RNA in our samples. Finally, we present a mathematical modelling framework that explicitly resolves active and dormant microbial groups, and apply this model, Micro‐Low Ice 1.1, to the glacier surface to simulate physiological state‐changes in the glacial microbial community under idealized (i) freezing, (ii) thawing, and (iii) freeze–thaw conditions. Our data indicates that on average 45%–62% of bacteria in glacial snow and ice are translationally active in‐situ during the summer melt period, with the remainder (38%–55%) of bacteria detected being dormant or dead. Ex‐situ incubations revealed that glacier microorganisms resumed translational activity within 24 hours of lab‐induced thawing following ∼6 months frozen at −20°C, suggesting that glacial microorganisms are able to respond rapidly to the dynamic and changing conditions of their environment. We therefore deduce that the biology and biogeochemistry of glacial surfaces are shaped by processes occurring over short (i.e., daily) timescales, and may be susceptible to change following the future alteration to glacial melt regimes caused by climate change.

## 2 MATERIALS AND METHODS

### 2.1 Study sites and sampling

We studied two glaciers: one in SE‐Greenland (Mittivakkat glacier) and other in Iceland (Langjökull) (Figure 1). Mittivakkat is a polythermal glacier separate from the Greenland Ice Sheet and is located on Ammassalik Island, SE Greenland. Langjökull is the second largest ice cap in Iceland, located in the west of the Icelandic interior. We focused on light‐absorbing particle‐containing snow and ice samples (following the description from Lutz et al., (2014)). In mid‐July 2019, we collected samples of ice (labelled MIT5) from the bare‐ice surface of Mittivakkat's ablation zone (65.691° N; 37.857° W, altitude 343 masl) (Halbach et al., 2022). A few weeks later, in early August 2019, we collected samples of both particle‐containing snow (IS19‐13) and ice (IS19‐14) from the surface of the north‐western edge of Langjökull's ablation zone. At Langjökull, sampling sites for snow (IS19‐13: 64.632° N; 20.508° W; altitude 1145 masl) and ice (IS19‐14: 64.635° N; 20.539° W; altitude 691 masl) were ~1.5 km apart. Analyses of the inorganic and organic chemical characteristics of a suite of snow and ice samples from Iceland as well as their 16S and 18S rRNA amplicon sequencing data (including IS19‐13 and IS19‐14) are described in Winkel et al., (2022).

Snow samples were collected from the top 1 cm of the snow surface. Ice samples were collected from surface scrapings (top 2–5 cm of the ice surface) using ethanol‐sterilized ice axes and spatulas. We scraped an ice surface area of ~1 m^2^ into a pile, which was mixed to homogenize the sample. Separate aliquots were collected into sterile WhirlPak® bags and sterile 50 ml Falcon® tubes for genomic analyses, BONCAT, pH, conductivity, and analyses of dissolved inorganic compounds. For dissolved organic carbon (DOC) analysis, separate samples were collected with sterile metal trowels into acid‐washed (~10% HCl, 24 h) and ashed (550°C, 6 h) glass jars.

Samples for molecular analyses, pH, conductivity, DOC, and dissolved inorganic compounds were thawed at room temperature on return to the field laboratory, and immediately processed once fully melted. For DNA and RNA analysis, 500 ml (IS19‐13), 250 ml (IS19‐14), and 500 ml (MIT5) of melted ice or snow were filtered through single use, sterile, 0.2 μm cellulose nitrate filters (Thermo Scientific Nalgene). Filters were removed from the units, folded with sterile forceps, and transferred into sterile 5 ml cryotubes that were immediately frozen and returned to the home laboratory in a liquid nitrogen cryo‐shipper and stored at −80°C until DNA/RNA extraction (Trivedi et al., 2022). A field blank, which consisted of autoclaved ultrapure water, was subjected to the same filtration process as described above in the field laboratory and the resulting filter was preserved and processed downstream following the same protocol as all other field samples. pH and conductivity were measured using portable conductivity and pH meters (HI 98129; Hanna instruments). For dissolved inorganic ions, thawed sample aliquots were filtered through 0.2 μm polycarbonate filters into either acid‐washed Nalgene® bottles (for cations, acidified upon return to the home institution with ultra‐pure nitric acid), or into 15 ml Falcon® tubes (for anions). Samples for DOC analysis were filtered using acid‐cleaned glass syringes, 0.7 μm ashed GF/F filters and acid‐washed metal filtration units, and the solutions were filtered directly into acid‐washed and ashed 40 ml borosilicate amber vials sealed with acid‐cleaned Teflon septas.

### 2.2 In‐situ BONCAT incubations

We used BONCAT to detect translationally active bacteria in‐situ on Langjökull, targeting both snow and ice supraglacial habitats. BONCAT is a non‐destructive click‐chemistry‐based method to visualize protein synthesis in uncultured environmental microorganisms in‐situ (Hatzenpichler et al., 2014). Samples for in‐situ BONCAT experiments were collected into small WhirlPak® bags, which were placed on plastic sheets on the surface of the glacier to melt at ambient air temperatures (5.4°C for snow and 7.4°C for ice samples). Following melting (30–60 min), 50 ml of each sample was aliquoted into a new WhirlPak® bag for incubations in triplicate. Each replicate was incubated in‐situ (i.e., on the snow/ice surface) with 500 μl of 500 μM HPG (Invitrogen™ Molecular Probes™ Click‐iT™ L‐Homopropargylglycin, Thermo Fisher Scientific) to a final concentration of 5 μM HPG. Incubations were allowed to react for 24 hours on the glacier surface after which they were quenched by the addition of glutaraldehyde (3% final concentration). The resulting samples were filtered onto 25 mm diameter 0.22 μm GTTP Isopore™ Membrane filters (Merck KGaA), left to dry and returned frozen and in the dark to the home lab. Sections of filters were placed onto clean glass slides and the HPG‐containing proteins were fluorescently labelled via a Cu(I)‐catalyzed azide–alkyne click reaction, thus marking cells on the filters that have undergone protein synthesis during the time of incubation (Hatzenpichler et al., 2016). A click‐reaction mixture was prepared from an Invitrogen™ Click‐iT® Cell Reaction Buffer Kit (Thermo Fisher Scientific), by mixing 155 μl of Milli‐Q® ultrapure water, 20 μl Click‐iT® cell reaction buffer (Component A, Reaction Buffer Kit), 4 μl of 100 mM copper sulphate aqueous solution (Component B, Reaction Buffer Kit), 0.5 μl of Invitrogen™ Alexa Fluor™ 488 Azide (Thermo Fisher Scientific), and 20 μl of Click‐iT® cell buffer additive (Component C, Reaction Buffer Kit). 25 μl of the click reaction mixture was added to the filter, before covering the filter with a coverslip. Slides were incubated in the dark for 30 min, and each filter was then washed twice in a succession of two baths of Milli‐Q ultrapure water. Once dried, the filters were mounted onto a clean glass slide with 5 μg ml^−1^ 4′ 6‐diamidino‐2 phenylindole (DAPI; Sigma–Aldrich) in Citifluor AF‐1 antifading solution (Electron Microscopy Sciences). Filters were then covered with a coverslip and slides were kept in the dark until microscopy analyses. We also performed in‐situ killed control BONCAT experiments by fixing thawed samples with glutaraldehyde (3% final concentration) before the in‐situ incubation with HPG. Following the incubation, all other steps were performed as described above. This control served to detect and measure the occurrence of non‐specific labelling (i.e., HPG incorporation by non‐active cells, thus creating an artefactual signal of translational activity).

### 2.3 Ex‐situ BONCAT incubations

Aliquots of samples IS19‐13 (snow) and IS19‐14 (ice) from Langjökull, and samples of ice from Mittivakkat glacier (MIT5), were also transported frozen to the home laboratory. Samples were stored frozen (−20°C) for approximately 6 months. Then samples were thawed and incubated in singlicate at 2°C under an 18 W fluorescent light for an initial incubation period ranging in duration from 1 hour to 38 days (hereafter referred to as the “pre‐incubation” period). Following this, samples were subject to a second incubation period at 2°C in the light, lasting 24 h following the addition of HPG (as above, final concentration 5 μM HPG) (hereafter referred to as the “HPG‐incubation” period), and quenched with glutaraldehyde (3% final concentration) as a fixing agent as described above. Here, we present total incubation time as the sum of the durations of the pre‐incubation period (prior to the addition of HPG) and the 24‐h HGP‐incubation period. Following the addition of glutaraldehyde, samples were filtered, and HPG‐containing proteins were fluorescently labelled as described above.

### 2.4 Microscopy and image analysis

Slides were analyzed using an epifluorescence microscope (Leica DM 2000) and images were acquired with a DFC 420C camera and a FI/RH (Leica) filter system following Winkel et al., (2018). Each filter was manually inspected for BONCAT signals, and representative images were taken for DAPI and BONCAT. Bacterial cells were counted manually from at least 20 random fields of view per slide. We always enumerated DAPI‐stained cells first, before switching to HPG, to not preferentially select fields of view for BONCAT‐positive (i.e., active) cells. Algal cells (determined by size and morphology) were rarely visible and were not included in counts.

### 2.5 Bacterial and eukaryotic diversity

The bacterial and eukaryotic diversity of Langjökull and Mittivakkat supraglacial habitats were characterized by 16S and 18S rRNA gene sequencing, respectively. DNA was extracted in a sterile laminar flow hood from all samples using the DNeasy PowerSoil DNA Isolation kits (QIAGEN GmbH). 16S rRNA genes were amplified using the bacterial primers 341F (5′‐CCTACGGGNGGCWGCAG) and 785R (5′‐GACTACHVGGGTATCTAATCC) spanning the V3‐V4 hypervariable regions. 18S rRNA genes were amplified using the eukaryotic primers 528F (5′‐GCGGTAATTCCAGCTCCAA) and 706R (5′‐AATCCRAGAATTTCACCTCT; (Cheung et al., 2010)) spanning the V4‐V5 hypervariable regions. Snow and ice samples from Langjökull (Sample IDs: IS19‐13 and IS19‐14, respectively) were sequenced on an Illumina MiSeq at the University of Bristol Genomics Facility using paired 2× 300 bp reads (v3 chemistry). Samples from Mittivakkat (Sample ID: MIT5) were sequenced on an Illumina MiSeq at the Department of Environmental Science (Environmental Microbiology) at Aarhus University using paired 2× 250 bp reads (v2 chemistry). Quality filtering (Table S1) was carried out using the dada2 pipeline (parameters: ‐‐p‐trim‐left‐f = 10, ‐‐p‐trim‐left‐r = 10). Due to the lower sequencing quality of the 2× 300 bp reads (IS19‐13 and IS19‐14) compared with the 2× 250 bp reads (MIT5), samples IS19‐13 and IS19‐14 were additionally truncated at 280 (R1) and 250 (R2) bp before merging. The amplicon sequence variants (ASV) in the denoised libraries were classified using classify‐sklearn and the SILVA database (silva‐138‐99‐nb‐classifier) (Quast et al., 2013) (Table S2). To avoid skewed relative abundance values, sequences matching chloroplast and mitochondrial DNA were excluded from the 16S dataset, as well as sequences matching “Bacteria” and “Archaea” from the 18S dataset. The most abundant algal ASVs were additionally subjected to manual BLAST to verify their taxonomic identity (Table S3). The resulting feature tables were rarefied to the lowest sample size (16S: 47900, 18S: 82900) and only ASVs with a minimum frequency count of 10 were retained. The processed Mittivakkat and Langjökull feature libraries were then merged and imported into R (v.3.6.0) (Team, 2021), where plots of the community compositions were created using the package “ggplot2” (Wickham, 2016).

### 2.6 Metatranscriptomics and determination of potentially active cells

RNA was extracted from all samples in a sterile laminar flow hood using the ZymoBIOMICS DNA/RNA Mini Kit (Zymo Research Europe GmbH) according to the manufacturer's protocol. TotalRNA libraries were prepared using the NEBNext Ultra II Direction RNA Library Prep kit (New England Biolabs GmbH) for Illumina. Extracted RNA concentrations were below the detection limit for all samples and therefore RNA was directly used in library preparation after extraction rather than being normalized to a standard concentration. The manufacturer's protocol was followed exactly using 12 μl of RNA directly (no dilution). The RNA was fragmented for 8 min, and 16 cycles were used for the library amplification. Libraries were indexed using the NEBNext Multiplex Oligos for Illumina. The resulting library concentrations and size distributions for IS19‐13 and IS19‐14 totalRNA were measured by qPCR and an Agilent 2100 Bioanalyzer, respectively. Equimolar pools were prepared for totalRNA, and quality checks showed the absence of primer dimers. The totalRNA pools were loaded on a V2‐flow cell for a 2×250 paired‐end sequencing run on an Illumina MiSeq according to the manufacturer's instructions. The library for sample MIT5 was measured and visualized on an Agilent 4150 Tapestation where concentration (measured on Qubit 4) and insert size were used to calculate the final molarity for sequencing. MIT5 was run as part of a larger pool on an Illumina NextSeq using the Mid Output v2.5 (300 cycles) kit.

Metatranscriptomic sequences were quality checked using FastQC v.0.11.8, and subsequently, quality filtered using the “illumina‐utils” package of tools developed by Eren et al., (2013). All FastQC reports were compiled for easier inspection and visualization using MultiQC v.1.9 (Ewels et al., 2016). For taxonomic inspection of the metatranscriptome (totalRNA) libraries, phyloFlash v3.4 (Gruber‐Vodicka et al., 2022) was used. Briefly, PhyloFlash annotates short sequences from metagenome and metatranscriptome data to profile SSU rRNA to the SILVA (v138) rRNA gene database (Quast et al., 2013). To avoid skewed relative abundance values, sequences matching chloroplast and mitochondrial DNA in the 16S rRNA RNA dataset were excluded. The resulting tables of taxonomic units (NTUs) were imported into R, and the data were manipulated and visualized using the tidyverse suite of R packages (Wickham et al., 2019).

### 2.7 Dissolved inorganic and organic compounds

The concentrations of dissolved inorganic major, minor, and trace elements in snow and ice samples were analyzed by inductively coupled plasma mass spectrometry (ICP‐MS; Thermo Fisher iCAPQc) following methods described in McCutcheon et al. (2021). The precision of the analyses varied between 2% and 5%. Inorganic anions were analyzed by ion chromatography (IC) using conductivity detection (ICS 3000, Dionex). DOC was analyzed by liquid chromatography‐organic carbon detector (LC‐OCD) where organic carbon was quantified by infrared detection of released CO_2_ after UV photo‐oxidation (185 nm) in a Gräntzel thin‐film reactor following Regenspurg et al. (2018).

### 2.8 Microbial model

**FIGURE 2:**
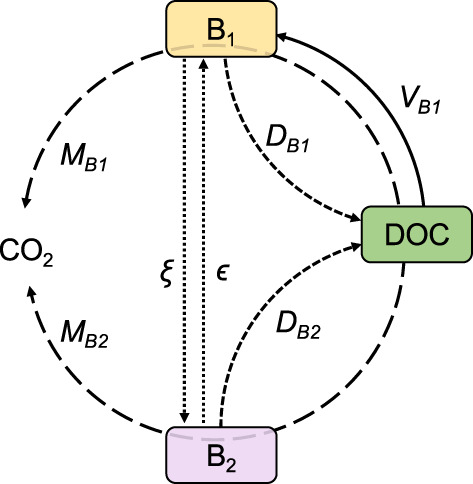
Conceptual model diagram. State variables: active biomass (*B*_*1*_), dormant biomass (*B*_*2*_), and dissolved organic carbon (*DOC*). Transfer functions: *V*_*B1*_ represents the growth of active biomass, *M*_*B1*_ and *M*_*B2*_ represent the maintenance of active and dormant biomass, respectively (i.e., the transformation of *DOC* to *CO*_*2*_ by *B*_*1*_ and *B*_*2*_), *D*_*B1*_ and *D*_*B2*_ stand for the mortality of active and dormant biomass respectively, *ξ* is the de‐activation of active to dormant biomass, and *ϵ* is the activation of dormant biomass to active biomass.

A microbially explicit process‐based ecosystem model, MicroLow Ice 1.1, is presented and implemented to simulate the transition of cells between active and inactive states during freezing and thawing. The microbial model implemented in this study divides microbial biomass, *B*, into two pools which are distinguished by their state of activity or dormancy: *B*_*1*_ represents active microorganisms and *B*_*2*_ represents dormant microorganisms. A system of coupled ordinary differential equations (Table S4) describes the transfers and transformations of these pools due to growth, maintenance (i.e., energy usage for processes other than growth), activation of the dormant fraction to the active fraction, deactivation of the active fraction to the dormant fraction, and death, according to the conceptual diagram shown in Figure 2. The state variables and initial conditions are listed in Table S5.

#### 2.8.1 Microbial dynamics

Our simplified model only considers the heterotrophic bacteria on the glacier surface. Therefore, we assume that the catabolic energy of microorganisms represented by *B* is derived from the degradation of *DOC*. The growth rate of the active biomass, *B*_1_, is dependent on *DOC* concentration, via Michaelis–Menten kinetics (Michaelis & Menten, 1913). Dormant cells (*B*_*2*_) are viable but must undergo activation before they are capable of growth. Dormancy requires that an organism (i) is not growing or dividing (i.e., has a reproductive rate equal to zero) and (ii) has a lower metabolic demand than when it is active (Lennon & Jones, 2011; Stolpovsky et al., 2011). Furthermore, dormant microorganisms may better endure inhospitable conditions and thus have a lower mortality rate than active microorganisms (Johnson et al., 2007; Lennon & Jones, 2011; Price & Sowers, 2004). All biomass pools (*B*_*1*_, *B*_*2*_) can die, utilize *DOC* to meet maintenance power requirements, and transition between active and dormant states.

The rate of change of active biomass (*B*_*1*_) is given by:

(1) $$\frac{{\partial B}_{1}}{\partial t}=V_{B1}-D_{B1}-\xi_{B1}+\epsilon_{B2}$$

where *t* is time (hours), *V*_*B1*_ represents the rate of new biomass growth, *D*_*B1*_ denotes the death rate of *B*_*1*_, *ξ*_*B1*_ corresponds to the deactivation of *B*_*1*_ into *B*_*2*_, and *ϵ*_*Bn*_ is the activation of *B*_*2*_ to *B*_*1*_.

Biomass growth is given as:

(2) $$V_{B1}=B_{1}\cdot v_{\max}\cdot \frac{\mathrm{DOC}}{K_{v}+\mathrm{DOC}}$$

where *B*_*1*_ is the concentration of active biomass (μg C l^−1^), *v*_*max*_ is the maximum growth rate of active biomass (hour^−1^), *DOC* is the concentration of dissolved organic carbon (μg C l^−1^), and *K*_*v*_ is the half‐saturation constant for microbial growth according to standard Michaelis–Menten kinetics (Michaelis & Menten, 1913).

Microbial death of biomass pool *B*_*n*_ (where *B*_*n*_ = *B*_*1*_ or *B*_*2*_) is given as:

(3) $$D_{\mathrm{Bn}}=\alpha_{B}\cdot B_{n}$$

where *α*_*Bn*_ is the mortality rate constant of biomass *B*_*n*_ (h^−1^).

The rate of change of dormant biomass (*B*_*2*_) is given by:

(4) $$\frac{{\partial B}_{2}}{\partial t}=-D_{B2}+\xi_{B1}-\epsilon_{B2}$$

where *D*_*B2*_ represents the death rate of *B*_*2*_, *ξ*_*B1*_ corresponds to the deactivation of *B*_*1*_ to *B*_*2*_, and *ϵ*_*Bn*_ is the activation of *B*_*2*_ to *B*_*1*_.

#### 2.8.2 Activation and deactivation

The model is based on the principle that microorganisms on glacial surfaces can take on different physiological states: from active and growing (*B*_*1*_), to dormant (*B*_*2*_). We prescribe that the deactivation and reactivation of biomass (i.e., the transitioning of states between *B*_*1*_ and *B*_*2*_) depends on temperature. In natural environments, the conditions that determine state‐changes depend on a multitude of environmental factors, including the potential supply of catabolic energy to the cells (e.g., Bradley et al. (2019)). However, these phenomena are not considered in this study.

The deactivation (ξ) of biomass *B*_*1*_ to *B*_*2*_ is given by:

(5) $$\xi_{B1}=\left(1-\theta_{S}\right)\cdot R_{S,D}\cdot B_{1}$$

where *R*_*S,D*_ is the specific rate of deactivation.

Similarly, the activation (*ϵ*) of biomass *B*_*2*_ to *B*_*1*_ is given by:

(6) $$\epsilon_{B2}=\theta_{S}\cdot R_{S,A}\cdot B_{2}$$

where *R*_*S,A*_ is the specific rate of activation.

*θ*_*S*_ is a function that accounts for the direction and rate of state‐change depending on temperature. It is based on the principle that if conditions in the environment are better than a certain threshold, there will be net activation of biomass, and vice‐versa. The function, from Stolpovsky et al. (2011), is adapted from Fermi–Dirac statistics:

(7) $$\theta_{S}=\frac{1}{e^{\left(\frac{-T+K_{S}}{{\mathrm{st}}_{S}\cdot K_{S}}\right)}+1}\;$$

where *K*_*S*_ is a threshold temperature for net activation/deactivation and *st*_*S*_ is a non‐dimensional parameter controlling the steepness of the sigmoidal function that determines the value and rate of change of *θ*_*S*_ for a given temperature range. There is a net deactivation of biomass (i.e., *B*_*1*_ to *B*_*2*_) under unfavourable conditions when the temperature is below *K*_*S*_ and thus *θ*_*S*_ < 0.5. Similarly, there is a net activation of biomass (i.e., *B*_*1*_ to *B*_*2*_) when the temperature rises above *K*_*S*_ and thus *θ*_*S*_ > 0.5.

#### 2.8.3 Maintenance

Both active and dormant cells carry out maintenance from exogenous (i.e., external substrate derived) catabolism. The rate of exogenous catabolism, *M*_*Bn*_, is described as:

(8) $$M_{\mathrm{Bn}}=B_{n}\cdot m_{\mathrm{Bn}}$$

where *m*_*Bn*_ is the specific maintenance power requirement of *B*_*n*_. Maintenance power requirements (*m*_*Bn*_) are described as a proportional carbon cost per unit of biomass per hour (i.e., similar to the mortality rate constant (*α*_*Bn*_)).

#### 2.8.4 Dissolved organic carbon dynamics

The rate of change of *DOC* is given as:

(9) $$\frac{\partial \mathrm{DOC}}{\partial t}=-\left(V_{B1}\cdot \frac{1}{Y}\right)-\sum M_{\mathrm{Bn}}+\sum D_{\mathrm{Bn}}$$

where *Y* represents the growth yield, *∑M*_*Bn*_ represents the sum of *DOC* consumption for maintenance by *B*_*1*_ and *B*_*2*_, and *∑D*_*Bn*_ represents the sum of the dead cells that contribute *DOC* from *B*_*1*_ and *B*_*2*_.

#### 2.8.5 Initial values and test data

We initialized our model using measurements carried out in the present study and from previously published data (Tables S5 and S6). We used an initial DOC concentration of 1200 μg C l^−1^, which is representative of the average measured DOC concentration at MIT5. We used an initial bacterial biomass carbon concentration of 0.55 μg C L^−1^, which is based on 5 × 10^4^ bacterial cells ml^−1^ and represents the measured concentration of bacteria occurring on glacier surface ice with a medium coverage of glacier algae, from the western margin of the Greenland Ice Sheet (Nicholes et al., 2019), and a bacterial biomass conversion factor of 11 fg C cell^−1^ (Anesio et al., 2010). The initial allocation of biomass between active and dormant states depends on the simulation carried out (see Section 2.8.6) and is summarized in Table S5.

#### 2.8.6 Implementation

We ran the model to explore activity and dormancy, and activation and deactivation according to ice surface melting and re‐freezing, reflecting idealized versions of the conditions occurring on Greenland glacier surfaces, as well as our ex‐situ BONCAT incubations. These scenarios are not intended to be truly representative of the natural glacier surface environment, but nevertheless provide a framework for exploring system dynamics, data‐model integration, and future model development.

We considered the following scenarios:

- FREEZE. This simulation assesses the response of an initially active microbial community to the onset of freezing. Temperature is prescribed at −3°C and the model is run for a period of 10 days.
- THAW. This simulation assesses the response of an initially dormant microbial community to the onset of melting. Temperature is prescribed at 0.5°C and the model is run for a period of 10 days.
- FREEZE–THAW. This simulation assesses the response of an initially dormant microbial community to periodic freezing and thawing. Temperature is prescribed to fluctuate above and below freezing: first at 0.5°C for 3 days, followed by −3°C for 3 days, then 0.5°C for 3 days, and finally −3°C for 1 day.

Model parameter values are summarized in Table S6. Prescribed temperatures for simulations (i)–(iii) were chosen to explore model dynamics when temperature varies above and below the threshold temperature for state‐change, *K*_*S*_.

#### 2.8.7 Numerical solution

The mathematical expressions described above and in Table S4 were implemented in the open‐source computing environment and programming language R (Team, 2022) using the deSolve package (Soetaert et al., 2010), which is freely available (http://www.r‐project.org/).

## 3 RESULTS

### 3.1 Microbial activity

**FIGURE 3:**
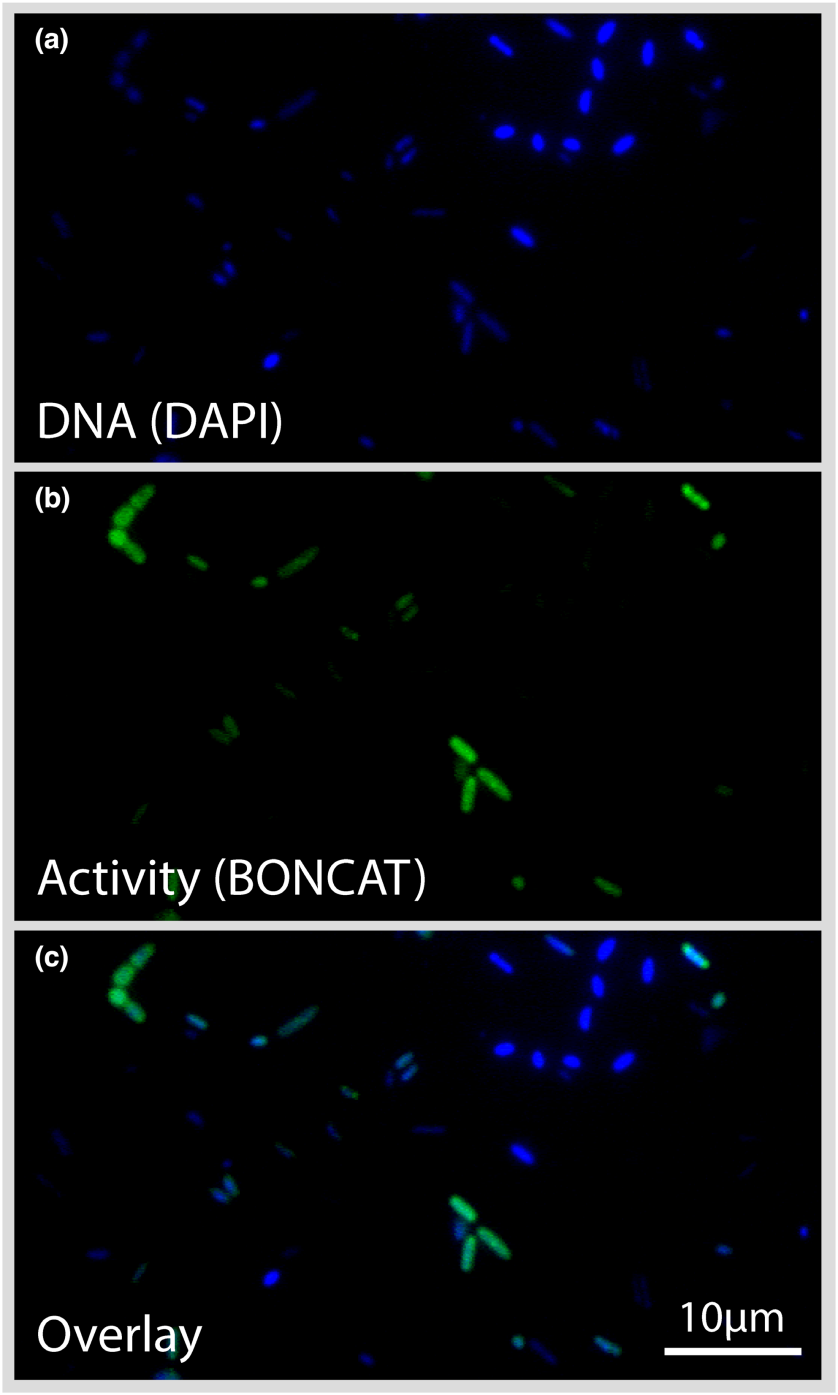
Epifluorescence microscopy. Single‐cell visualization of translational activity observed for bacteria from the ice sample MIT5 from Mittivakkat glacier. Panels from top to bottom: (a) DAPI staining of DNA in blue; (b) protein synthesis‐active cells via BONCAT in green; and (c) an overlay showing active (green) and inactive (blue) cells.

We found that HPG was actively incorporated by bacteria incubated in‐situ on the glacier surface and ex‐situ in the lab. After HPG incubations, some cells acquired a distinct green fluorescence signal corresponding to the azide dye addition to the BONCAT label (Figure 3; Figure S1).

Image analyses from the incubated samples revealed blue DAPI‐stained cells, and green BONCAT+ cells (Figure 3; Figure S1). An overlay of the two images shows both BONCAT+ and BONCAT− cells in relation to all DAPI‐stained cells. DAPI+ and BONCAT+ (blue+/green+) cells are those engaged in nascent protein synthesis and are classified as active, while DAPI+ and BONCAT− (blue+/green−) cells are classified as inactive/dormant or dead. Both translationally active as well as inactive or dead cells were observed consistently across all fields of view for all samples (except for killed controls and blanks, where cells did not acquire fluorescence following the click reactions).

**FIGURE 4:**
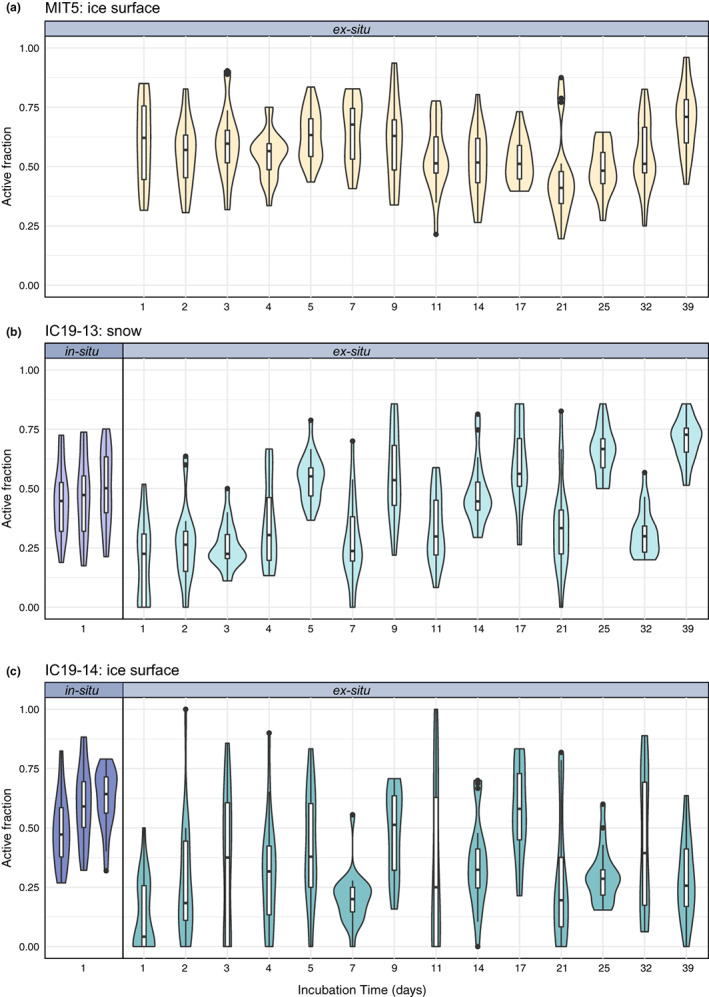
Microbial activity in in‐situ 24‐h incubations and ex‐situ longer‐term laboratory incubations. The active fraction of bacteria (as determined via BONCAT) in (a) Mittivakkat ice samples, (b) Langjökull snow samples, and (c) Langjökull ice samples. Both in‐situ (i.e., on the glacier surface) incubations (in triplicate), and an ex‐situ time‐series of laboratory incubations at 2°C after thawing from ∼6 months storage at −20°C (in singlicate), are shown. Incubation time (days) represents the sum of the incubation period prior to the addition of HPG (“pre‐incubation”) and the 24‐h incubation with HPG (“HPG‐incubation”). The outer shape of the violin plot represents the kernel density distribution of the data, where wider sections indicate a higher density of data.

In all in‐situ incubations from Langjökull, approximately half of the bacterial cells were found to be translationally active (on average 52%, ±16%, 1 SD) (Figure 4). The active fraction of cells within a single incubation ranged from 17% to 88% for Langjökull snow and ice. The mean active fraction of bacteria in the snow was 47% (±16%). The variability between replicate incubations was low and not significantly different from each other (one‐way ANOVA, *p* = .493, Table S7). The mean active fraction of cells in ice was greater than in snow (57%, ±15%, *p* = .001), and there was greater variability between replicates for the ice habitat (one‐way ANOVA, *p* = .011).

For ex‐situ incubations (which tested microbial activity following thawing of frozen samples), all samples from all three habitats (Mittivakkat ice and Langjökull snow and ice), including those incubated with HPG immediately (1 hour) post‐thaw, contained active cells (Figure 4). On average, 60% (±17%) of cells in the Mittivakkat ice sample were active during the shortest (1 day) ex‐situ incubation, whereas fewer cells were active in the ex‐situ snow and ice samples from Langjökull incubated for 1 day (on average 19% of cells in snow and 13% of cells in ice). The proportion of active cells in Mittivakkat ice samples and Langjökull snow samples did not show an increase in 3 days following thaw (one‐way ANOVA, *p* = .573 and *p* = .259, respectively), whilst the active fraction of cells in Langjökull glacier ice was found to increase from an average of 13% at 1 day post‐thaw to 35% at 3 days post‐thaw (35%) (*p* = .022; Figure S2; Table S7).

Longer‐term ex‐situ incubations were conducted for up to 39 days. The activity was overall higher among cells from Mittivakkat glacier ice samples than in Langjökull snow (*p* < .001) and ice (*p* < .001). The mean active fraction of cells in ex‐situ incubations of Mittivakkat ice samples was 45–70% (SD = 14%), in Langjökull snow it was 26–70% (SD = 14%), and in Langjökull ice it was 13–57% (SD = 22%). Across all ex‐situ incubations for samples from Langjökull, the active fraction of cells in the snow (IS19‐13) was found to be higher than in ice (IS19‐14) (*p* < .001).

For Langjökull, we compared the active fraction of cells in‐situ with ex‐situ incubations following thaw (Figure 4). For one to four days following thawing (ex‐situ), the active fraction of cells in snow appeared to remain lower than in‐situ levels (*p* < .001). However, five days after thaw (ex‐situ), the fraction of active cells in Langjökull snow samples (54%, ±10%) appeared to reach in‐situ activity levels (47%, ±16%) (*p* = .069). Nevertheless, variation in the active‐fraction of cells across singlicate incubations and incubation times of 1–39 days was high and recurrently deviated above and below in‐situ activity levels. For samples of ice from Langjökull, the active fraction of bacteria reached in‐situ levels or higher two days after thawing.

### 3.2 Microbial community composition

**FIGURE 5:**
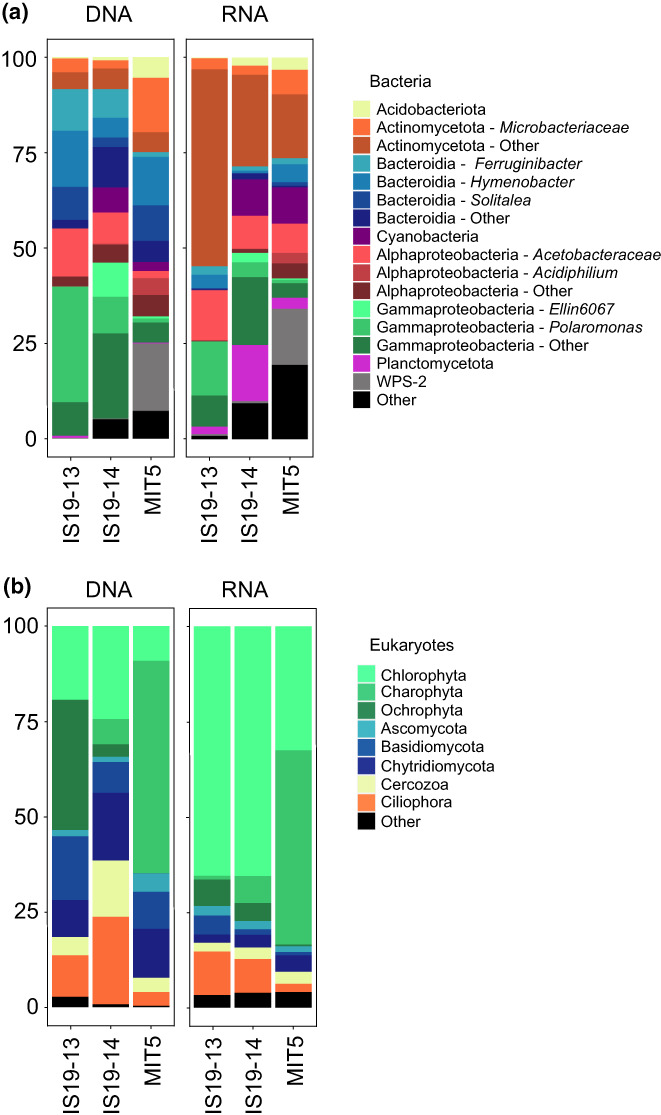
Microbial community composition and activity. Stacked bar‐plots showing (a) bacterial community composition (DNA, based on 16S rRNA gene relative abundance, %) and the active community (RNA, based on 16S rRNA gene expression from total RNA, %). The most abundant ASVs are shown. The remaining ASVs within one class are combined into phylum/class. ASVs within one class share broadly the same hue. (b) Eukaryotic community composition (DNA, based on 18S rRNA gene relative abundance) and the active community (RNA, based on 18S rRNA gene expression from total RNA, %).

For bacteria, there was a high degree of similarity on all phylogenetic levels between the snow (IS19‐13) and ice (IS19‐14) samples from Langjökull (Figure 5a). The most abundant bacterial phyla were Pseudomonadota (snow: 54%, ice: 54%), Bacteroidota (snow: 37%, ice: 26%), and Actinomycetota (snow: 8%, ice: 8%). Cyanobacteria were not found in the snow, but comprised 7% of all sequences in the ice and were predominantly represented by the family *Leptolyngbyaceae* (6%) (Table S2). Within the Pseudomonadota, Gammaproteobacteria (snow: 39%, ice: 41%), and Alphaproteobacteria (snow: 15%, ice: 13%) were most abundant. The Gammaproteobacteria were mostly represented by the genus *Polaromonas* (snow: 30%, ice: 10%). Alphaproteobacteria were mostly represented by the family *Acetobacteraceae* (snow: 14%, ice: 10%). Within the Bacteroidota, the genera *Hymenobacter* (snow: 15%, ice: 6%) and *Ferruginibacter* (snow: 12%, ice: 12%) were dominant. Actinomycetota were mostly represented by the families *Microbacteriaceae* (snow: 4%, ice: 2%), *Nocardiaceae* (snow: 3%, ice: 1%), and *Sporichthyacea* (ice: 3%, snow: 0%). The most abundant bacterial phyla in ice from Mittivakkat (MIT5) were also Bacteroidota (29%), Actinomycetota (20%), Pseudomonadota (19%), and in contrast to Langjökull, we also found WPS‐2 (17%). Bacteroidota were mostly represented by the genera *Hymenobacter* (16%) and *Solitalea* (9%). Actinomycetota mostly comprised the family *Microbacteriaceae* (14%). Within the Pseudomonadota, Alphaproteobacteria (12%) were most abundant with *Rhodanobacter* (2%) being the dominant genus, followed by Gammaproteobacteria (7%) with *Acidiphilium* (4%) as the most abundant genus.

The eukaryotic community composition varied fairly evenly on the phylum level across the Langjökull snow and ice samples (Figure 5b). The algal composition comprised Chlorophyta, Ochrophyta, and Phragmoplastophyta (Charophyta). Ochrophyta were only highly abundant (34%) in the snow sample IS19‐13 and were entirely made up of the genus *Hydrurus* (Table S3). Chlorophyta was abundant in all three habitats sampled (IS19‐14: 24%, IS19‐13: 19%, MIT5: 9%). Within the Chlorophyta, the most abundant families were *Trebouxiophyceae* (IS19‐13: 8%, IS19‐14: 12%, MIT5: 5%) and *Chlorophyceae* (IS19‐13: 10%, IS19‐14: 10%, MIT5: 4%). The two most abundant ASVs corresponding to *Chlorophycaea* shared the highest similarity with several *Chloromonas* species and were thus assigned to *Chloromonas* spp. (a: IS19‐13: 1%, IS19‐14: 3%, MIT5: 0%; b: IS19‐13: 4%, IS19‐14: 0%, MIT5: 0%). ASVs matching *Chlainomonas* sp. (IS19‐13: 0%, IS19‐14: 3%, MIT5: 1%) and *Chlamydomonas nivalis* (IS19‐13: 0%, IS19‐14: 1%., MIT5: 2%) were also present in lower abundances.

Phragmoplastophyta (Charophyta) were highly abundant in the ice sample from Mittivakkat glacier (56%), in contrast to the samples from Langjökull (IS19‐13: 0%, IS19‐14: 7%). The Phragmoplastophyta were entirely comprised of Zygnematophyceae. One ASV matching *Ancylonema nordenskiöldii* was only present at MIT5 (38%), whereas the second ASV (also matching *A. nordenskiöldii*) could also be found in low abundances in the other samples (MIT5: 11%, IS19‐14: 2%, IS19‐13: 0%). *Ancylonema alaskanum* was present in both ice samples (MIT5: 6%, IS19‐14: 3%) albeit in low abundance.

The fungal community composition comprised mostly of Basidiomycota, Chytridiomycota, and Ascomycota. Basidiomycota were most abundant in IS19‐13 (17%), followed by MIT5 (10%) and IS19‐14 (8%). Chytridiomycota showed the highest relative abundance in IS19‐14 (18%) followed by MIT5 (13%) and IS19‐13 (10%). Ascomycota were most abundant in MIT5 (5%) and below 2% in the other two samples. In addition, Ciliophora (IS19‐13: 11%, IS19‐14: 23%, MIT5: 4%) and Cercozoa (IS19‐13: 5%, IS19‐14: 15%, MIT5: 4%) showed a higher relative abundance in all three samples.

### 3.3 Taxonomic classification of active bacteria and eukaryotes

Relative abundance bar plots of the recovered 16S rRNA genes from total RNA reveal diverse communities of potentially active bacteria. We found that the taxonomic classifications for both DNA and RNA were broadly similar for bacteria: the organisms that were generally abundant in DNA were also detected in the RNA reads. Specifically, there was a high representation of sequences belonging to Actinomycetota, Pseudomonadota, and Plancomycota among total RNA sequences in Langjökull snow and ice, and a high representation of cyanobacteria in RNA reads from Langjökull and Mittivakkat ice. Notably, Bacteroidota were not strongly represented in the RNA reads, despite their high relative abundance in the DNA sequences across all samples. For eukaryotes, the taxonomic classifications for DNA and RNA were broadly similar, with a high representation of DNA sequences belonging to Chlorophyta in all samples. In particular among the Chlorophyta, *Chlorophyceae* were particularly strongly represented in Langjökull snow and ice (27–32% of total 18S rRNA genes extracted from total RNA), as well as *C. nivalis* (6%–19% of total 18S rRNA genes extracted from total RNA in all samples), and *Prasiolales* in Langjökull snow and ice (7%–11% of total 18S rRNA genes extracted from total RNA). *A. nordenskiöldii*, which were found to be dominant among the Charophyta in Mittivakkat glacier ice, were strongly represented in 18S rRNA genes extracted from total RNA from Mittivakkat.

### 3.4 Dissolved inorganic and organic compounds

We found that snow and ice from Langjökull and ice from Mittivakkat had largely similar pH, conductivity, and concentrations of dissolved Mg^2+^ and Ca^2+^ ions (Table S8). Snow from Langjökull had higher concentrations of sea‐salt derived major ions (Cl^−^, Na^+^), as well as Mn^2+^, whilst glacier ice, especially from Mittivakkat, had higher concentrations of DOC, Fe^2+^, K^+^, and Zn^2+^. NO_3_^−^ and SO_4_^2−^ were below detection limits (50 ppb) in all samples.

### 3.5 Modelling active and dormant microorganisms on the glacier surface

We simulated the activity of the glacial surface microbial community using a process‐based mathematical model that considers the physiological transitions between the active and dormant states of microorganisms, and their interaction with glacial DOC. The model was applied to an ice surface habitat under highly idealized scenarios of (i) freezing, (ii) thawing, and (iii) freeze–thaw cycles. We account only for heterotrophic bacteria – which comprise most bacteria found to inhabit glacier surfaces (Lutz et al., 2017), and prescribed an initial biomass concentration of 0.55 μg C L^−1^ (approximately 5 × 10^4^ cells ml^−1^; (Anesio et al., 2010; Nicholes et al., 2019)).

**FIGURE 6:**
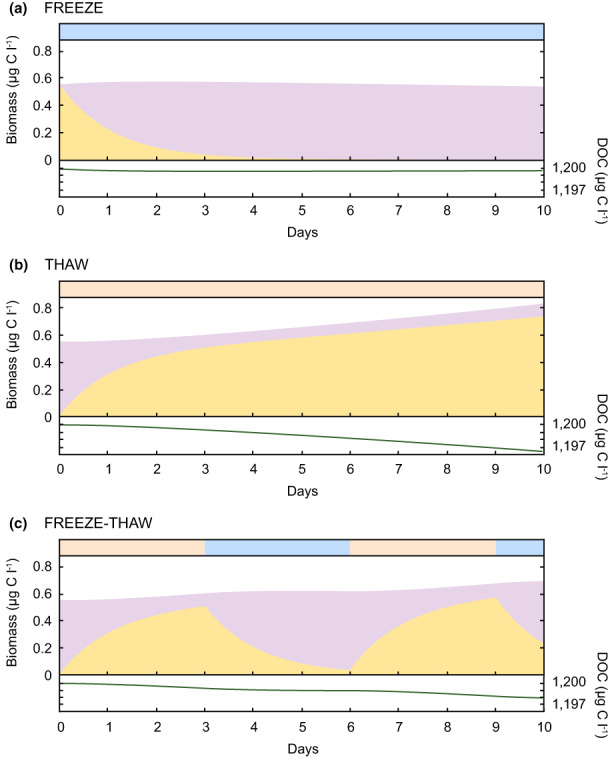
Modelling of active and dormant microorganisms. Simulations of active (*B*_*1*_, yellow) and dormant (*B*_*2*_, lilac) biomass, and DOC concentration, during idealized (a) freeze, (b) thaw, and (c) freeze–thaw conditions on a glacier surface. Red and blue bars at the top of each panel indicate the frozen (−3.0°C, blue) or thawed (0.5°C, red) state of the simulated glacier surface environment.

We found that freezing (*Scenario i: FREEZE;* T < K_S_) triggered a rapid transition of the active microbial community to a dormant state (Figure 6a). The majority (>60%) of the community transitioned into a dormant (i.e., non‐growing) state within 24 hours, and beyond 5 days less than 1% of the community remained active. We also observed minimal biomass growth and DOC consumption during the freezing scenario, and the majority of catabolic activity (from dormant rather than active microorganisms) was put towards maintenance rather than new biomass synthesis.

We found that thawing (*Scenario ii: THAW*; T > K_S_) triggered a rapid transition of initially dormant microorganisms to an active state (Figure 6b) – in particular during the first day of the simulation, where the active proportion rose from 0% (day 0) to 55% (day 1). We observed a net increase in biomass from 0.55 μg C L^−1^ (day 0) to 0.83 μg C L^−1^ (day 10), with microbial growth sustained throughout the simulation, and some consumption of DOC.

The third scenario (*Scenario iii: FREEZE–THAW*) examined the response of an initially dormant community to a thaw period (3 days), followed by a freeze (3 days), thaw (3 days), and final freeze (1 day) (Figure 6c). Like Scenarios (i) and (ii), we observed the active fraction of organisms rapidly increasing following thaw, and decreasing during freezing periods. We also observed more biomass growth and DOC consumption during thaw periods than during freezing. During freezing periods, catabolic activity went mainly towards maintenance, whereas during thaw periods, catabolic activity went towards both growth and maintenance.

## 4 DISCUSSION

Our data indicate that glacier surface microbial communities are comprised of both active and inactive organisms, which are capable of state‐switching on timescales similar to the freeze–thaw cycles experienced on glacier surfaces. Our sampling was conducted in the ablation zones of Mittivakkat and Langjökull, during mid (Greenland) to mid/late (Iceland) summer. This is, in both cases, at the height of the melt season, when liquid water (at ~0.1°C) is widespread on the glacier surface, coinciding with high bacterial and algal activity (Hamilton & Havig, 2017; Nicholes et al., 2019). At the same time, bacterial and algal production may be inhibited by high levels of photosynthetically active radiation (Halbach et al., 2022; Williamson et al., 2020; Yallop et al., 2012) and UV (Morgan‐Kiss et al., 2006), low nutrient availability (Edwards et al., 2013; Hamilton & Havig, 2017; Lutz, Anesio, Field, & Benning, 2015; Wadham et al., 2016), and diurnal freeze–thaw cycles (Cook et al., 2016). Dormancy may be a critical ecological strategy used by glacial microorganisms to survive these stresses (Greening et al., 2019; Roszak & Colwell, 1987; Sussman & Douthit, 1973). We found, using BONCAT, that a high proportion (on average 45%–62%, per incubation) of the bacteria in Langjökull snow and ice samples were translationally active in‐situ (Figure 4). The recovery of RNA from our samples is also suggestive of an active microbial community in Langjökull and Mittivakkat snow and ice at the time of sampling. Concurrently, we also found that a significant fraction (38%–55%) of cells in all samples were in a metabolically inactive state, that is, dormant or dead. We used our sequencing results to evaluate possible active and inactive taxa. We found a dominance of Pseudomonadota, Bacteroidota, and Actinomycetota in the total and active bacterial communities of Langjökull and Mittivakkat. These organisms are known to be well‐adapted to environmental fluctuations that characterize glacial surface environments (Cameron et al., 2012; Edwards et al., 2011; Gokul et al., 2016; Larose et al., 2010; Lutz et al., 2017). Pseudomonadota, which made up 19%–54% of DNA reads and 19%–36% of total RNA reads, have previously been shown to thrive on oligotrophic glacier surfaces (Eiler et al., 2003), and may be well suited to maintaining some metabolic activity despite the low carbon and nutrient concentrations of the snow and ice habitats sampled (Table S8). Notably, Bacteroidota – which were abundant in all snow and ice samples (>25% of DNA reads), were poorly represented (3%–8%) by the 16S rRNA genes extracted from total RNA. Although this could be a result of PCR bias from amplicon sequencing (Browne et al., 2020; Tremblay et al., 2015) compared to the metatranscriptomics, this finding may also be suggestive of Bacteroidota being disproportionately less active than other bacteria in the snow and ice samples from Langjökull and Mittivakkat. The detection of Bacteroidota in our samples compares well with previous data from the same glaciers in Iceland and Greenland (Lutz et al., 2017; Lutz, Anesio, Edwards, & Benning, 2015). Similarly, it has previously been shown that Bacteroidota are well suited to relatively nutrient‐scarce glacial surface habitats (Hamilton & Havig, 2017). Some members of Bacteroidota are known to have the ability to degrade high molecular weight organic C compounds, including polysaccharides (Cottrell & Kirchman, 2000), and are potentially utilizing algal necromass as a carbon and energy source (Teeling et al., 2012) enabling their growth and proliferation through the snowpack and across the ice surface. Additionally, Bacteroidota are known to be dominant in cryoconite holes (Edwards et al., 2013, 2014), which, although were not sampled as part of this study, are dynamic and hydrologically connected to adjacent supraglacial habitats (Cameron et al., 2020). Although Bacteroidota may be passively dispersed throughout the glacier surface ecosystem, they may be better suited to (and therefore more metabolically active in) cryoconite holes rather than the bare ice or snow surface. Other groups including Actinomycetota, Planctomycota, and Cyanobacteria, were found to be disproportionately more active than Bacteroidota, based on the high proportion of 16S rRNA sequences from total RNA reads that belonged to these groups.

Eukaryotes are important players in the supraglacial snow and ice ecosystem and are closely linked to bacterial biodiversity (Nicholes et al., 2019). Although our assessment of microbial activity on glacier surfaces with BONCAT does not include representation from eukaryotic algae, analysis of 18S rRNA sequences extracted from total RNA suggests that there was activity among the eukaryotic microorganisms present on the glacier surfaces. We found a prevalence of Chlorophyta (in particular *Chloromonas* sp. and *C. nivalis*, and *Trebouxiophyceae*) and Ochrophyta (*Hydrurus*) among the total and active eukaryotes in our samples – which is typical of red snow and yellow snow algal blooms, respectively (Lutz et al., 2014; Lutz, Anesio, Edwards, & Benning, 2015; Lutz, Anesio, Field, & Benning, 2015; Müller et al., 2001; Takeuchi, 2013). The dominance of Phragmoplastophyta (Charophyta) in ice from Mittivakkat glacier, in particular ASVs matching *A. nordenskiöldii* and *A. alaskanum*, is typical for glacier surfaces across the Arctic, as well as the Antarctic (Ling & Seppelt, 1993; Lutz et al., 2017, 2018; Remias et al., 2009; Takeuchi, 2013; Uetake et al., 2010; Yallop et al., 2012). Glacial snow and ice algal blooms provide a source of algal necromass and organic matter to bacterial communities, which can be used as a carbon and energy source – and this potentially supported bacterial activity at the time of sampling. Previous work has also shown that snow‐pack‐dwelling bacteria have the capacity to degrade high molecular weight organic compounds (proteins, lipids, carbohydrates, lignin) via enzymes including lipase, protease, amylase, −galactosidase, cellulase, and/or lignin modifying enzyme (Antony et al., 2016), suggestive of links between the algal primary producers and the largely heterotrophic bacterial community. A number of studies have shown a high abundance of Pseudomonadota in snow and ice that is associated with algal blooms (Lutz et al., 2014; Lutz, Anesio, Edwards, & Benning, 2015; Lutz, Anesio, Field, & Benning, 2015). As well as algae, we found a high prevalence of fungi in snow and ice from Langjökull and Mittivakkat, in particular, Chytridiomycota, which have been found previously in supraglacial environments (Brown et al., 2015; Lutz, Anesio, Edwards, & Benning, 2015; Naff et al., 2013; Perini et al., 2019) and are thought to be key players in nutrient recycling (Ibelings et al., 2004), perhaps facilitating bacterial production.

We showed via BONCAT (Figure 4) that microbial communities resume activity rapidly following thaw, even after entombment in a frozen state for ∼6 months. We found significant labelling and thus translational activity among cells within 24 hours of thawing ice samples from Mittivakkat, and within several days of thawing snow and ice samples from Langjökull. We suggest therefore that microbial communities on glacier surfaces are capable of rapid state‐switching in response to fluctuating environmental conditions, such as diurnal freeze–thaw cycles which occur commonly on glacier surfaces during the summer melt period. We found a significantly higher active fraction of cells in the 24 hours following the thaw from Mittivakkat ice samples than from snow and ice collected from Langjökull. In addition, Mittivakkat samples had a higher active fraction of bacteria than Langjökull samples over the course of the thaw experiment. The apparent rapid responsive switching of cells at Mittivakkat could potentially be attributed to a community that is better adapted to dealing with fluctuating environmental conditions including freeze–thaw cycles, harsh winters, resource scarcity, and nutrient limitation.

Ice surfaces are dynamic environments that are both hydrologically connected and temporally variable, and accordingly, glacial surface microbiota are continually responding to environmental fluctuations (Cameron et al., 2020). The high variability of the active fraction of cells – particularly from Langjökull ice samples incubated under ex‐situ conditions (Figure 4), is perhaps reflective of this. Dormancy in microbial communities is induced in response to unfavourable changes in an environment (“responsive switching”), as well as via “spontaneous switching,” that is, a stochastic re‐initiation of metabolic activity. Theoretically, responsive switching is generally favoured in fluctuating environments, whereas spontaneous switching should be adaptationally favoured under stable conditions (Kussell & Leibler, 2005). On a glacier, microorganisms would likely have an evolutionary advantage to be responsive to fluctuating environmental conditions (temperature, presence of liquid water, and light), as well as starvation and resource limitation. Dormancy is likely to be of particular importance in high‐latitude regions that are subject to distinct seasonal cycles, in which microorganisms must contend with harsh winters, yet be in good stead to take advantage of favourable environmental conditions including sunlight, liquid water, and nutrients that characterize the spring and summer periods. Based on our observations that (i) a sizeable fraction (on average 38–55% per incubation) of glacial bacterial cells are dormant (or dead) in‐situ and (ii) snow and ice bacteria rapidly resume activity following thaw, we suggest that responsive dormancy may be of critical importance to the survival of microorganisms among glacial settings, and like other natural environments (Jones & Lennon, 2010), glacier surface microbial communities may be structured largely by environmental cues that trigger dormancy and activity. In addition, glacier surface microbial communities receive aeolian inputs from neighboring environments that might contain bacteria that are poorly suited to the glacial environment (Bowers et al., 2012; Harding et al., 2011; Pearce et al., 2016; Xiang et al., 2009) – and thus bacteria derived from atmospheric deposition might comprise some fraction of the inactive (or dead) community detected here.

Attempts to quantify bacterial activity in glacial settings have typically relied on measurements of bulk (rather than cell‐specific) reaction rates, such as the incorporation of ^14^C or ^3^H‐leucine labels as a proxy for heterotrophic activity (Anesio et al., 2009; Karl et al., 1999; Nicholes et al., 2019; Rassner et al., 2016). Measurements of the activity (e.g., carbon fixation and heterotrophic production) of individual cells in glacial environments are lacking, however, cell‐specific activity can be estimated by combining bulk rate measurements with measurements of cell abundance – intrinsically assuming that every organism is equally active. Such cell‐specific rates have since been used to inform upscaling calculations and model parameter values (Bradley et al., 2015, 2017; Bradley, Arndt, et al., 2016; Nicholes et al., 2019). Importantly, data presented here clearly show that, in fact, some microorganisms are much more active than others. It is therefore important to understand and quantify the active and dormant fraction of the microbial community so that the true range of activity (and inactivity) of glacial microorganisms is captured in empirical studies of glacial habitats, and can be accurately incorporated into models (Bradley, Anesio, & Arndt, 2016; Stibal et al., 2017), including those which explicitly resolve microorganisms and their transitions between active and dormant states (Bär et al., 2002; Blagodatsky & Richter, 1998; Bradley et al., 2018, 2019; Ingwersen et al., 2008; Panikov, 1995; Stolpovsky et al., 2011).

Our ecological model simulates physiological state‐changes in the glacial microbial community informed by our experimental data and data available in the literature. Many of the model's central parameters (e.g., mortality rate constants, *α*_*Bn*_; maintenance constant, *m*_*Bn*_; half‐saturation for growth, *K*_*v*_; and steepness of state‐change dependency, *st*_*S*_) have never been experimentally or theoretically determined for glacier surfaces. Thus, we associate a high degree of uncertainty to both parameter values and the model output – including the rates of growth and death, the production and consumption of DOC, and the rapidity of state‐transitions. Our modelling framework is designed to be flexible to allow for additional complexity to be introduced, such as (i) resolving different groups of microorganisms with varying rates of transitioning between active and dormant states, (ii) resolving additional biomass groups such as eukaryotic photosynthetic algae, and (iii) integration with albedo and melt models (e.g., (Cook, Hodson, Gardner, et al., 2017; Cook, Hodson, Taggart, et al., 2017)). Further, our model assumes that microorganisms on glacial surfaces transition between active and dormant states depending on the temperature of the environment. In reality, the activity or inactivity of an individual cell depends on other environmental conditions including resource availability and environmental harshness (e.g., UV exposure, predation, and viral infection). These controls could also be included in future iterations and applications of this model.

The responsiveness of the microbial community to changing environmental conditions is a key variable in predicting whether glacial ecosystems and the biogeochemical cycles they drive are sensitive to anthropogenic climate changes – including air temperature warming, the lengthening of the melt season, and the frequency of short‐term periodic thaw events. The resumption of activity of microorganisms after only a relatively short thaw period (1 day) following freezing (∼6 months) is illustrative of the capacity for microbial communities to respond rapidly to periodic thawing – even if this thaw is of short duration (e.g., winter melt events). Winter warming and rain events are likely to become more frequent occurrences as the Arctic responds to anthropogenic climate change (Graham et al., 2017; Post et al., 2019). This may have knock‐on consequences for the glacial microbial community. For example, Ganey et al. (2017) demonstrated that the addition of water to a snowpack over a 2‐month period over summer increased algal counts in the snow by 48%. However, there is a lack of studies on the direct and indirect consequences of changing the timing, duration, and extent of precipitation and melt events across the surface of Arctic glaciers on microbial communities. Moreover, alteration to the activity of microbial communities on glacier surfaces as a result of anthropogenic climate change may impact associated microbially driven biogeochemical cycles. In both our ex‐situ BONCAT experiments and modelling, we demonstrate that the glacial microbial community is sensitive to thaw events on the timescale of days. The high sensitivity of the resuscitation phase of dormancy to temperature and melt, as demonstrated here, suggests that sustained periods of melt are not necessary for significant alteration to microbial community dynamics. In fact, even short (1 day) periodic melt events could induce the reactivation of cells among a largely dormant population – thus bringing about a significant change to the functioning of the glacial ecosystem. Our evidence for rapid state‐switching suggests that glacier and snow habitats effectively become active biogeochemical reactors whenever conditions are such that liquid water is available to the microbial community. Accordingly, we find it plausible that future alterations to the timing and extent of melt on Arctic glaciers and ice sheets will affect the dynamics of both the active and dormant fractions of the glacial microbial community. It follows that any changes to microbial ecosystems could exert cascading effects on carbon and nutrient cycles in glacial systems (Margesin & Collins, 2019) and adjacent ecosystems (Cauvy‐Fraunié & Dangles, 2019; Irvine‐Fynn et al., 2021; Stibal et al., 2020).

## 5 CONCLUSIONS

Dormancy is a bet‐hedging strategy that is likely to be of particular importance to microorganisms inhabiting glacier surfaces and other cryo‐environments. Glacier surfaces are subject to frequent (i.e., hourly to daily) freeze–thaw conditions, as well as distinct seasonal cycles during which microorganisms must contend with prolonged periods of cold and darkness lasting several months or longer. Moreover, microorganisms in glacial environments must be capable of reactivation during periods of favourable environmental conditions (e.g., abundant sunlight and liquid water during spring and summer periods). Here, we have taken the first steps to quantify the prevalence of active and dormant microorganisms in Arctic supraglacial habitats. We found that microbial communities in our glacial snow and ice samples were comprised of active cells (on average 45%–62%), but also contained inactive or dead individual cells (on average 38%–55%). We provide evidence that the diverse assemblages of microorganisms on glacier snow and ice surfaces responded to environmental conditions by switching between active and inactive metabolic states, which may be critical to enabling their persistence through adverse conditions. There is a need to further investigate the links between microbial state‐switching and resource availability and nutrient limitation on glacier surfaces. Crucially, our results suggest that glacial microorganisms are able to respond to short melt‐events occurring on the timescale of hours to days – which is sufficiently short that periodic melting on glacier surfaces potentially impacts the functioning of glacial ecosystems and biogeochemical cycles. Enhanced winter warming is predicted to become more prevalent as a result of future climate change and could therefore bring about ecological changes to glaciers. Progress in measuring, mechanistically capturing, and quantifying state‐switching and the associated biogeochemical consequences will be fundamental in understanding the ecological role of dormancy in glacial ecosystems, and the potential impacts of climate change. Studying the dynamic nature of microbial habitats on glaciers and ice sheets is critical to understanding them as refugia for microbial life, analogue environments of potentially habitable icy worlds, and regulators of regional and global biogeochemical cycles.

## CONFLICT OF INTEREST

The authors declare no competing interests.

## Supporting information


Appendix S1

Table S1

Table S2

Table S3

## Data Availability

Data and model code are available in Appendix S1.
