# Supplementary material for: Active and dormant microorganisms on glacier surfaces
Source: Geobiology. 2022 Nov 30;21(2):244–61. doi: 10.1111/gbi.12535 (PMC10099831; doi:10.1111/gbi.12535)
Supplement: Supplementary file 1 — Appendix S1 [file GBI-21-244-s004.docx]

Supplementary Information

**Active and dormant microorganisms on glacier surfaces**

James A. Bradley^1,2^, Christopher B. Trivedi^2^, Matthias Winkel^2,3^, Rey Mourot^2,4^, Stefanie Lutz^2^, Catherine Larose^5^, Christoph Keuschnig^4^, Eva Doting^6^, Laura Halbach^6^, Athanasios Zervas^6^, Alexandre M. Anesio^6^, Liane G. Benning^2,4^.

^1^ Queen Mary University of London, London, UK

^2^ GFZ German Research Center for Geosciences, Potsdam, Germany

^3^ Bundesanstalt für Risikobewertung (BfR), Germany

^4^ Freie University Berlin, Berlin, Germany

^5^ Environmental Microbial Genomics, Université de Lyon, Ecully Cedex, France

^6^ Aarhus University, Environmental Science, iClimate, Roskilde, Denmark

**Data Availability**

*Model code:* MicroLow Ice 1.1 is written and can be executed in the free open-source computing environment and programming language R, which is available for download on the web (http://www.r-project.org/). A package named “MICROLOW_ICE_1.1_SOURCE”, containing the source code and a ReadMe guide is available at https://github.com/jbradley8365/MICROLOW_ICE_1.1_SOURCE. The entire “MICROLOW_ICE_1.1_SOURCE” folder should be copied to a local computer such that the directory is: “/Users/jamesbradley/Documents/RFolder/MICROLOW_ICE_1.1_SOURCE/” or the folder paths contained in ‘execute_MICROLOW_ICE_1.1.R’ script should be modified (setwd, path, pathte) according to the location where the folder is copied to.

*Sequencing data:* Sequencing data is available via NCBI under the following accession numbers:

**BioProject PRJNA657180**

IS19-13 DNA: SAMN15830840

IS19-14 DNA: SAMN15830841

IS19-13 RNA: SAMN26570441

IS19-14 RNA: SAMN26570444

**BioProject PRJNA893676**

MIT5 DNA: SAMN31525315, SAMN31525316

MIT5 RNA: SAMN31525317

*Bioinformatic pipelines:* <https://github.com/jbradley8365/Geobiology_Bradley_dormancy_data_processing>

**Supplementary Table 1 - Sequencing statistics**

Provided as separate Excel document.

**Supplementary Table 2 - Prokaryotic community composition**

Provided as separate Excel document.

**Supplementary Table 3 - Eukaryotic community composition**

Provided as separate Excel document.

**Supplementary Table 4. Microbial model formulation.**

| **Fundamental balance equations** |  |
| --- | --- |
| Rate of change of *B_1_* | $\frac{{\partial B}_{1}}{\partial t}=V_{B1}-D_{B1}-\xi_{B1}+\epsilon_{B2}$ |
| Rate of change of *B_2_* | $\frac{{\partial B}_{2}}{\partial t}=-D_{B2}+\xi_{B1}-\epsilon_{B2}$ |
| Rate of change of *DOC* | $\frac{\partial DOC}{\partial t}=-\left( V_{B1}\cdot\frac{1}{Y} \right)-\sum M_{Bn}+\sum D_{Bn}$ |
| **Microbial growth and death** |  |
| Growth of *B_1_* | $V_{B1}=B_{1}\cdot v_{max}\cdot\left( \frac{DOC}{K_{v}+DOC} \right)$ |
| Death of biomass *B_n_* | $D_{Bn}=\alpha_{Bn}\cdot B_{n}$ |
| Total death | $\sum D_{Bn}=D_{B1}+D_{B2}$ |
| **Deactivation and activation** |  |
| Deactivation of biomass (*B_1_→B_2_*) | $\xi_{B1}=\left( 1-\theta_{S} \right){\cdot R}_{S,D}\cdot B_{1}$ |
| Activation of biomass (*B_2_→B_1_*) | $\epsilon_{B2}={\theta_{S}\cdot R}_{S,A}\cdot B_{2}$ |
| Function to determine the direction of change of state (i.e. active to dormant, and vice-versa) | $\theta_{S}=\frac{1}{e^{\left( \frac{-T+K_{S}}{{st}_{S}\times K_{S}} \right)}+1}$ |
| **Maintenance** |  |
| Maintenance (*B_n_*) | $M_{Bn}={B_{n}\cdot m}_{Bn}$ |
| Total maintenance | $\sum M_{Bn}=M_{B1}+M_{B2}$ |

**Supplementary Table 5. State variables and initial values.**

| **State Variable** | **Description** | **Initial value (µg C l^-1^)** | | |
| --- | --- | --- | --- | --- |
|  |  | (i) FREEZE | (ii) THAW | (iii) FREEZE-THAW |
| *B_1_* | Active biomass | 0.55 | 0 | 0 |
| *B_2_* | Dormant biomass | 0 | 0.55 | 0.55 |
| *DOC* | Dissolved organic carbon | 1200 | 1200 | 1200 |

**Supplementary Table 6. Parameters.**

| Parameter | Description | Units | Nominal value | Reference |
| --- | --- | --- | --- | --- |
| *v_max_* | Maximum growth rate of *B_1_* | Hour^-1^ | 0.048 | Nicholes et al. (2019); Anesio et al. (2010) |
| *K_v_* | Half-saturation constant for growth of *B_1_*, based on *DOC* concentration | µg C l^-1^ | 8000 | Fitted |
| *Y* | Growth yield of *B_1_* | Unitless | 0.2 | Fitted |
| *m_B1_* | Maintenance demand of *B_1_* | Hour^-1^ | 0.0005 | Fitted |
| *m_B2_* | Maintenance demand of *B_2_* | Hour^-1^ | 0.00005 | Fitted |
| *α_B1_* | Mortality rate constant of *B_1_* | Hour^-1^ | 0.004 | Fitted |
| *α_B2_* | Mortality rate constant of *B_2_* | Hour^-1^ | 0.0004 | Fitted |
| *st_S_* | Steepness of state-change dependency | Unitless | 0.2 | Stolpovsky et al. (2011) |
| *K_S_* | Threshold temperature for state-change | °C | 0.1 | Fitted |
| *R_S,D_* | Rate constant for deactivation | Hour^-1^ | 0.04 | Fitted |
| *R_S,A_* | Rate constant for activation | Hour^-1^ | 0.04 | Fitted |

**Supplementary Table 7. Statistical significance tests for BONCAT incubations**

| **In-situ incubations; One way ANOVA** | | | |  | |
| --- | --- | --- | --- | --- | --- |
| Samples | P-value | | F-value | |  |
| IS19-13 (in-situ) & IS19-14 (in-situ) | 0.001 | | 11.25 | |  |
| IS19-13 replicates (x3) | 0.493 | | 0.717 | |  |
| IS19-14 replicates (x3) | 0.011 | | 4.856 | |  |
| **IS19-14 replicates; Tukey multiple pairwise-comparisons** | | | |  | |
| Samples | | P-value | |  | |
| IS19-14: R2-R1 | | 0.050 | |  | |
| IS19-14: R3-R1 | | 0.012 | |  | |
| IS19-14: R3-R1 | | 0.867 | |  | |
| **In-situ and ex-situ treatments; One way ANOVA** | | | |  | |
| Samples | | P-value | | F-value | |
| IS19-13 in-situ & 1-day incubation | | <0.001 | | 45.52 | |
| IS19-13 in-situ & 2-day incubation | | <0.001 | | 27.04 | |
| IS19-13 in-situ & 3-day incubation | | <0.001 | | 33.22 | |
| IS19-13 in-situ & 4-day incubation | | 0.005 | | 8.188 | |
| IS19-13 in-situ & 5-day incubation | | 0.069 | | 3.39 | |
| IS19-13 in-situ & 6-day incubation | | <0.001 | | 22.01 | |
| IS19-14 in-situ & 1-day incubation | | <0.001 | | 13.92 | |
| IS19-14 in-situ & 2-day incubation | | 0.012 | | 6.697 | |
| IS19-14 in-situ & 3-day incubation | | 0.097 | | 2.823 | |
| **First three days of ex-situ incubation; One way ANOVA** | | | |  | |
| Sample | | P-value | | F-value | |
| IS19-13 | | 0.259 | | 1.384 | |
| IS19-14 | | 0.022 | | 4.082 | |
| MIT5 | | 0.573 | | 0.563 | |
| **Ex-situ incubations (all time-periods); one way ANOVA** | | | |  | |
| Samples | | P-value | | F-value | |
| IS19-13 & MIT5 | | <0.001 | | 89.53 | |
| IS19-14 & MIT5 | | <0.001 | | 176.8 | |

**Supplementary Table 8. Chemical data.**

DOC is given in μg C l^-1^; Cl^-^, NO_3_^-^, SO_4_^2-^, Na^2+^, K^+^, Mg^2+^, Ca^2+^, Fe^2+^, Mn^2+^, and Zn^2+^ are given in ppb; bdl below detection limit; LOD’s for cations: Mg^2+^, Ca^2+^ = 3 ppb; K^+^, Na^2+^, 1 ppb, Fe^2+^ = 0.5 ppb, Mn^2+^, Zn^2+^ = 0.02 ppb, LOD’s for anions: Cl^-^, NO_3_^-^, SO_4_^2-^, DOC = 50 μg C l^-1^. For MIT5, A-C indicate triplicate samples.

| Sample ID | Habitat | pH | Conductivity (µs cm^-1^) | DOC | Cl^-^ | NO_3_^-^ | SO_4_^2-^ | Na^2+^ | K^+^ | Mg^2+^ | Ca^2+^ | Fe^2+^ | Mn^2+^ | Zn^2+^ |
| --- | --- | --- | --- | --- | --- | --- | --- | --- | --- | --- | --- | --- | --- | --- |
| IS19-13 | Snow | 5.55 | 1.93 | 300 | 282 | bdl | bdl | 184 | 6 | 6 | 24 | <1 | 1.3 | 0.7 |
| IS19-14 | Ice | 5.73 | 1.42 | 600 | bdl | bdl | bdl | 9 | 3 | 3 | 15 | 2 | 0.6 | 1.3 |
| MIT5-A | Ice | 5.13 | 1.58 | 1700 | bdl | bdl | bdl | 15 | 44 | 8 | 23 | 27 | 0.3 | 17 |
| MIT5-B | Ice | 5.68 | 1.45 | 500 | bdl | bdl | bdl | 34 | 42 | 9 | 22 | 47 | 0.3 | 13 |
| MIT5-C | Ice | 5.68 | 1.43 | 1400 | bdl | bdl | bdl | 41 | 48 | 13 | 25 | 76 | 0.4 | 17 |


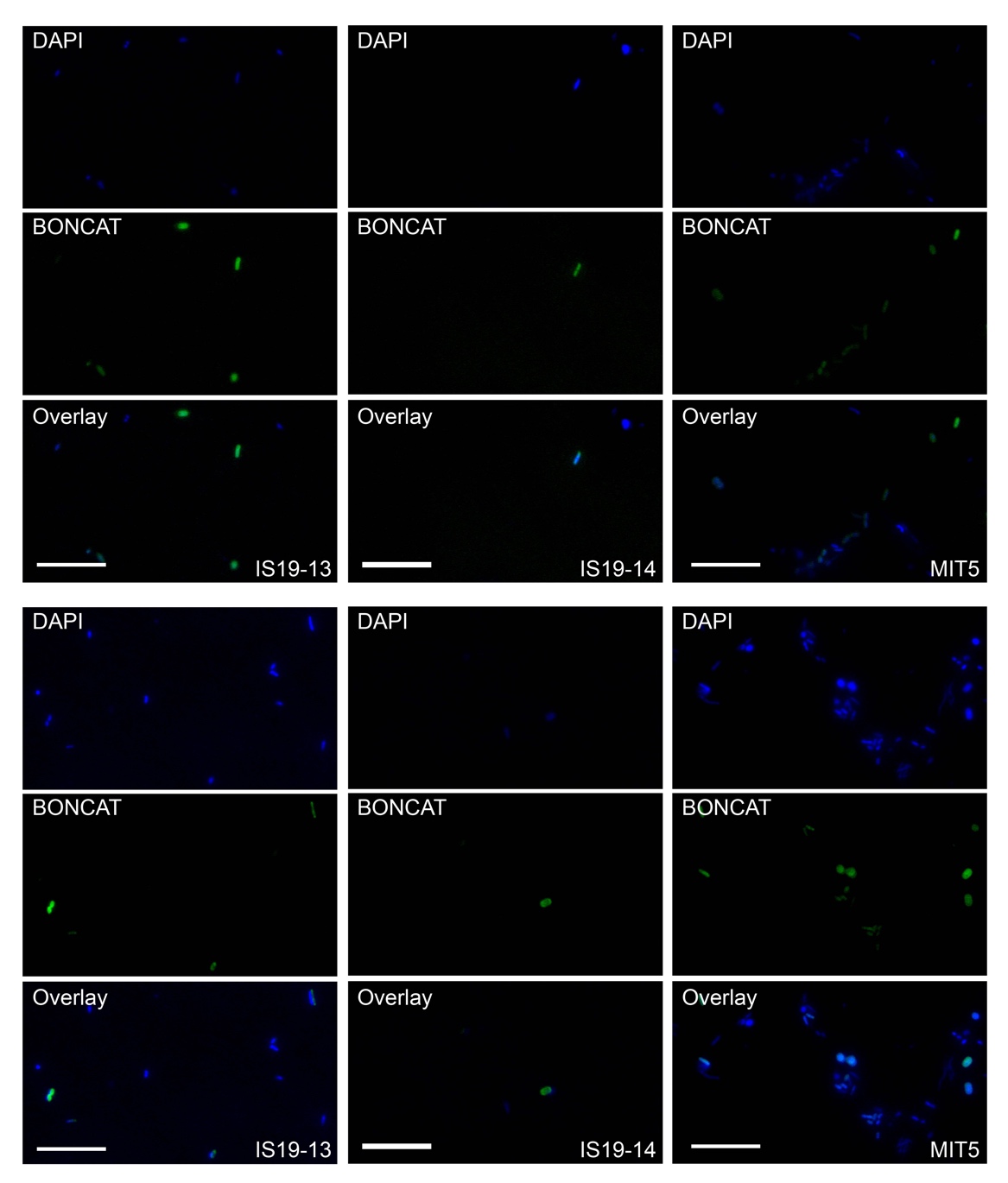


**Supplementary Figure 1. Epifluorescence microscopy**. Single-cell visualization of translational activity observed for bacteria from Langjökull snow (IS19-13), ice (IS19-14) and Mittivakkat glacier ice (MIT5). DAPI staining shows cells in blue; BONCAT shows protein synthesis-active cells in green; and an overlay shows active (green) and inactive (blue) cells.

**
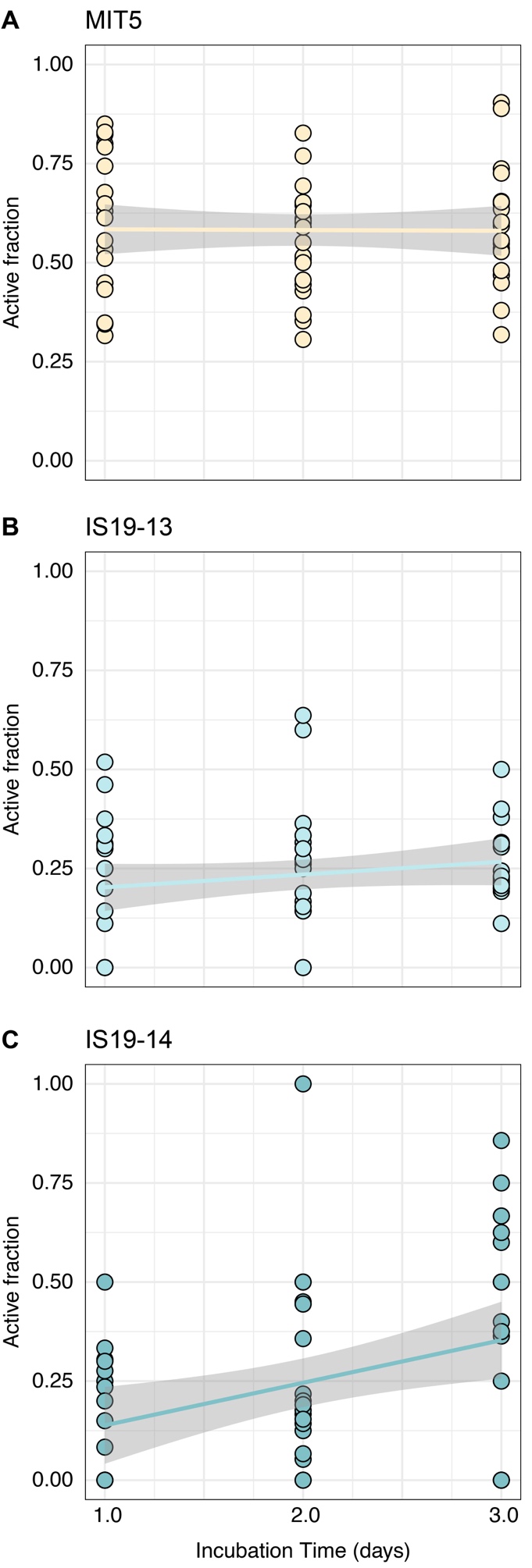
**

**Supplementary Figure 2. Microbial activity in ex-situ laboratory incubations.** The active fraction of cells (as determined via BONCAT) in (A) Mittivakkat ice samples (MIT5), and Langjökull (B) snow (IS19-13) and (C) ice (IS19-14) samples, during 2°C laboratory incubations following >6 months at -20°C, over the first three days of ex-situ incubations.

**Supplementary References**

Anesio, A. M., Sattler, B., Foreman, C., Telling, J., Hodson, A., Tranter, M., et al. (2010). Carbon fluxes through bacterial communities on glacier surfaces. *Ann. Glaciol*. 51, 32–40.

Nicholes, M. J., Williamson, C. J., Tranter, M., Holland, A., Poniecka, E., Yallop, M. L., et al. (2019). Bacterial Dynamics in Supraglacial Habitats of the Greenland Ice Sheet. *Front. Microbiol*. 10, 1366.

Stolpovsky, K., Martinez-Lavanchy, P., Heipieper, H. J., Van Cappellen, P., and Thullner, M. (2011). Incorporating dormancy in dynamic microbial community models. *Ecol. Modell.* 222, 3092–3102. DOI: 10.1016/j.ecolmodel.2011.07.006.
